# Disparities in Anticoagulant Therapy Initiation for Incident Atrial Fibrillation by Race/Ethnicity Among Patients in the Veterans Health Administration System

**DOI:** 10.1001/jamanetworkopen.2021.14234

**Published:** 2021-07-28

**Authors:** Utibe R. Essien, Nadejda Kim, Leslie R. M. Hausmann, Maria K. Mor, Chester B. Good, Jared W. Magnani, Terrence M. A. Litam, Walid F. Gellad, Michael J. Fine

**Affiliations:** 1 Center for Health Equity Research and Promotion, VA Pittsburgh Healthcare System, Pittsburgh, Pennsylvania; 2 Division of General Internal Medicine, University of Pittsburgh School of Medicine, Pittsburgh, Pennsylvania; 3 Department of Biostatistics, University of Pittsburgh Graduate School of Public Health, Pittsburgh, Pennsylvania; 4 Centers for Value-Based Pharmacy Initiatives and High-Value Health Care, UPMC Health Plan, Pittsburgh, Pennsylvania; 5 Department of Medicine, University of Pittsburgh School of Medicine, Pittsburgh, Pennsylvania

## Abstract

**Importance:**

Atrial fibrillation is a common cardiac rhythm disturbance causing substantial morbidity and mortality that disproportionately affects racial/ethnic minority groups. Anticoagulation reduces stroke risk in atrial fibrillation, yet studies show it is underprescribed in racial/ethnic minority patients.

**Objective:**

To compare initiation of anticoagulant therapy by race/ethnicity for patients in the Veterans Health Administration (VA) system with atrial fibrillation.

**Design, Setting, and Participants:**

This retrospective cohort study included 111 666 patients within the VA system with incident atrial fibrillation between January 1, 2014, and December 31, 2018. Data were analyzed between December 1, 2019, and March 31, 2020.

**Exposures:**

Any anticoagulation was defined as receipt of warfarin or direct-acting oral anticoagulants, apixaban, dabigatran, edoxaban, or rivaroxaban.

**Main Outcomes and Measures:**

Initiation of any anticoagulation (or direct-acting oral anticoagulant therapy in those who initiated any anticoagulation) was examined within 90 days of an index atrial fibrillation diagnosis.

**Results:**

Our final cohort comprised 111 666 patients (109 386 men [98.0%] and 95 493 White patients [85.5%]; mean [SD] age, 72.9 [10.4] years). A total of 69 590 patients (62.3%) initiated any anticoagulant therapy, varying 10.5 percentage points by race/ethnicity (*P* < .001); initiation was lowest in Asian (52.2% [n = 676]) and Black (60.3% [n = 6177]) patients and highest in White patients (62.7% [n = 59 881]). Among anticoagulant initiators, 45 381 (65.2%) used direct-acting oral anticoagulants, varying 7.2 percentage points by race/ethnicity (*P* < .001); initiation was lowest in Hispanic (58.3% [n = 1470]), American Indian/Alaska Native (59.8% [n = 201]), and Black (60.9% [n = 3763]) patients and highest in White patients (66.0% [n = 39 502). Compared with White patients, the odds of initiating any anticoagulant therapy were significantly lower for Asian (adjusted odds ratio [aOR], 0.82; 95% CI, 0.72-0.94) and Black (aOR, 0.90; 95% CI 0.85-0.95) patients. Among initiators, the adjusted odds of direct-acting oral anticoagulant initiation were significantly lower for Hispanic (aOR, 0.79; 95% CI, 0.70-0.89), American Indian/Alaska Native (aOR, 0.75; 95% CI, 0.57-0.99), and Black (aOR, 0.74; 95% CI 0.69-0.80) patients.

**Conclusions and Relevance:**

This cohort study found that in patients with incident atrial fibrillation managed in the VA system, race/ethnicity was independently associated with initiating any anticoagulant therapy and direct-acting oral anticoagulant use among anticoagulant initiators. Understanding the reasons for these treatment disparities is essential to improving equitable atrial fibrillation management and outcomes among racial/ethnic minority patients treated in the VA system.

## Key Points

**Question:**

Among patients with atrial fibrillation treated in the Veterans Health Administration system, are there racial/ethnic differences in the initiation of anticoagulation therapy?

**Findings:**

In this cohort study of a nationally retrospective cohort of 111 666 patients with atrial fibrillation treated from 2014 to 2018, Black and Asian patients were less likely to initiate any anticoagulation therapy. Among initiators, Black, Hispanic, and American Indian/Alaska Native patients were less likely to initiate direct oral anticoagulants.

**Meaning:**

In the Veterans Health Administration system, a national, integrated health system with improved access to medications through a uniform national drug formulary, racial and ethnic disparities appear to persist in atrial fibrillation management.

## Teaser

This cohort study uses data from the Veterans Health Administration system to compare rates of initiation of anticoagulant therapy by race/ethnicity.

## Introduction

Atrial fibrillation (AF) is the most common cardiac rhythm disorder in the United States, affecting up to 6 million adults.^1,2^ The Veterans Health Administration (VA) oversees the largest integrated health system in the country, treating nearly 1 million individuals with AF.^3^ Atrial fibrillation increases the risk of all-cause mortality and is associated with high rates of cardiovascular morbidity, including stroke,^4,5,6,7^ hospitalization, and outpatient and pharmacy costs.^8,9^

Oral anticoagulation (OAC) for nonvalvular AF reduces stroke risk by up to 70% and is the standard of care in patients with moderate to severe stroke risk.^10,11,12^ For decades, warfarin was the only OAC available for stroke prevention in patients with AF, but its use was challenging owing to frequent blood monitoring requirements, dosing adjustments, and food and drug interactions.^13,14^ A newer class of direct oral anticoagulants (DOACs) with substantially fewer management challenges was initially approved by the US Food and Drug Administration in 2010.^15,16,17^ Over time, DOACs have demonstrated superior clinical outcomes, cost effectiveness, safety, and adherence compared with warfarin.^18,19^ Consequently, recent cardiovascular practice guidelines have endorsed DOACs as first-line anticoagulant therapy for eligible patients with AF.^20^

Despite the effectiveness of OAC, there are racial/ethnic inequities in the initiation of such therapy.^21,22^ In prior analyses using insurance claims and clinical registries, racial/ethnic minority patients with AF were less likely than White individuals to be treated with any form of OAC, and DOACs in particular, even when controlling for patient sociodemographic and clinical characteristics.^23,24,25^ The gravity of these disparities, described in non-VA care settings, are amplified by the fact that racial/ethnic minority individuals with AF have higher rates of stroke and mortality than White individuals.^26,27,28,29^ The VA system provides an advantageous environment to examine treatment disparities, as it facilitates access to primary and specialty care and provides medications to its enrollees through a uniform national drug formulary. Our primary aim was to compare OAC initiation by race/ethnicity for patients with new-onset AF managed in the VA system. We also assessed patterns of OAC initiation by race/ethnicity over a time frame coinciding with the increased availability of DOACs in VA.

## Methods

For this cohort study, we compared OAC initiation by race/ethnicity in the Race, Ethnicity and Anticoagulant Choice in Atrial Fibrillation (REACH-AF) cohort. We developed the REACH-AF cohort as a national, retrospective cohort of patients enrolled in the VA system with incident, nonvalvular AF from 2010 through 2018. The institutional review board at the VA Pittsburgh Healthcare System approved the study and granted a waiver of informed consent, as data in the cohort were deidentified. We followed the Strengthening the Reporting of Observational Studies in Epidemiology (STROBE) reporting guideline.

### Data Sources

We used administrative and clinical data from the VA Corporate Data Warehouse to identify the cohort and create all relevant study variables. The Corporate Data Warehouse contains information on outpatient and inpatient clinical encounters, including patient sociodemographic details, diagnosis codes, VA clinic stop codes, and all prescribed medications dispensed by VA pharmacies. We used the University of Wisconsin School of Medicine and Public Health’s Neighborhood Atlas to link patient residence to the area deprivation index, a validated neighborhood-level socioeconomic score.^30,31^

### Cohort Eligibility Criteria

**Figure 1.**
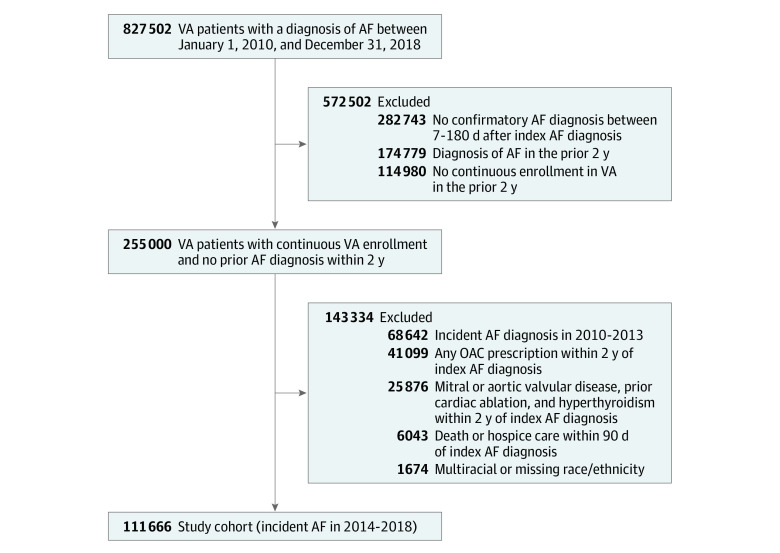
Identification of the Study Sample Among 827 505 patients with an index atrial fibrillation (AF) diagnosis between 2010 and 2018, we identified 255 000 patients with continuous VA enrollment, no prior AF diagnosis in the previous 2 years, and a confirmatory AF diagnosis within 180 days after their index diagnosis. After applying additional exclusion criteria, the study cohort included 111 666 patients with incident AF from 2014 through 2018. All exclusions were performed sequentially. OAC indicates oral anticoagulation.

We used established *International Classification of Diseases, Ninth Revision *(*ICD-9*) and *International Statistical Classification of Diseases and Related Health Problems, Tenth Revision* (*ICD-10*) diagnosis codes for AF from VA outpatient clinical encounters to identify unique patients with an initial diagnosis of AF from January 1, 2010, to December 31, 2018.^3,32^ We used the date of the first detected AF diagnosis code to represent the index (initial) diagnosis. To restrict the cohort to patients with incident AF treated in the VA system, we excluded those with any prior outpatient diagnosis of AF or who were not enrolled in the VA system in the 2 years prior to their index diagnosis. We required all patients to have 1 or more outpatient confirmatory diagnoses of AF within 7 to 180 days of their index diagnosis (Figure 1).^32,33^ Among 827 502 patients in the REACH-AF cohort with an index AF diagnosis between 2010 and 2018, 255 000 had continuous VA enrollment, no AF diagnosis in the 2 years prior, and a confirmatory AF diagnosis between 7 and 180 days after the index AF event.

To restrict the cohort to nonvalvular AF, we excluded patients with a diagnosis code for any form of aortic or mitral valvular disease, repair, or replacement prior to their index AF diagnosis.^3,33^ We excluded patients with diagnoses for cardiac ablation or hyperthyroidism and those receiving any OAC therapy within 2 years prior to the index AF diagnosis.^33^ Consistent with prior studies, we excluded patients who died or were enrolled in hospice care within 90 days after their index AF diagnosis.^32,33^ We excluded those missing race/ethnicity (0.6% [633 of 113 340 patients]) and multiracial individuals (0.9% [1041 of 113 340 patients]) given the small number and heterogeneity of these patients. Finally, we restricted our assessment to individuals with an index AF diagnosis between January 1, 2014, and December 31, 2018, when all 3 of the most commonly prescribed DOACs (apixaban, dabigatran, and rivaroxaban) were available on the national VA drug formulary.^3^ The *ICD-9* and *ICD-10* diagnosis codes used to determine cohort eligibility are shown in eTable 1 in the Supplement. After removing those with valvular heart disease and other exclusionary medical conditions, with prior anticoagulant therapy, in hospice care or who died within 90 days after the index diagnosis, with missing race/ethnicity or who were multiracial, or with an AF diagnosis in the years preceding the broad availability of DOAC therapy in the VA system (2010-2013), 111 666 patients comprised the study sample (Figure 1).

### Study Outcomes

The primary outcome was initiation of any form of OAC, defined as the first outpatient filled prescription for warfarin or DOAC (ie, apixaban, dabigatran, edoxaban, or rivaroxaban) within 90 days of an index AF diagnosis. For those who initiated any OAC, we assessed initiation of DOAC or warfarin within this time frame.

### Independent Variable and Baseline Patient, Health Care Professional, and Facility Covariates

Based on a health disparities research framework and prior AF disparities studies,^23,34,35^ we examined patient, health care professional, and facility-level characteristics considered potential drivers or confounders of the association between our primary independent variable (ie, race/ethnicity) and study outcomes. We used VA administrative data on race/ethnicity, supplemented by data from the Centers for Medicare and Medicaid Services when missing, to define this variable as non-Hispanic White (ie, White), non-Hispanic Black (ie, Black), Hispanic, Asian, or American Indian/Alaska Native (AI/AN).^36,37^

We identified additional baseline patient sociodemographic characteristics, including a categorized variable for age at index diagnosis, sex, and VA priority group (groups 1-8), which conveys patients’ level of eligibility for VA services given their financial means, service-connected health conditions, and disabilities (lower groups have increased eligibility for VA benefits).^38^ We assessed geographic census region and level of rurality based on each patients’ residence.^36^ We used patient residential 9-digit zip code (zip + 4) to link to the area deprivation index, a comprehensive ranking of neighborhoods by socioeconomic disadvantage using factors from the domains of income, education, employment, and housing quality.^30^ We categorized this index into quintiles using percentile rankings of all US neighborhoods from least (1st percentile) to most (100th percentile) disadvantaged. We also coded the calendar year of the index AF diagnosis.

We assessed patient clinical variables using inpatient and outpatient diagnosis codes within the 2 years before an index AF diagnosis.^33,39^ The comorbid conditions were heart failure, hypertension, diabetes, vascular disease, prior stroke, and renal disease. We classified body mass index into 5 groups reflecting levels of underweight, normal weight, and overweight.^40^ We used the validated CHA_2_DS_2_VASc prediction rule (a score composed of points for congestive heart failure; hypertension; age ≥75 years; diabetes mellitus; prior stroke, transient ischemic attack, or thromboembolism; vascular disease; age 65-74 years; and sex category [female]) to quantify the 1-year stroke risk in AF, categorized as low (score 0-1), moderate (score 2-4), and high (score >4) risk.^41^ We also assessed variables comprising the validated HAS-BLED prediction rule (a score composed of points for hypertension, abnormal kidney/liver function, stroke, bleeding history or predisposition, labile international normalized ratio, age >65 years, and use of drugs or alcohol) of 1-year bleeding risk in AF that were not otherwise captured in our analyses, including history of prior bleeding, liver disease, and use of medications predisposing to bleeding (eg, antiplatelets, nonsteroidal anti-inflammatory drugs).^42,43^ The *ICD-9* and *ICD-10* diagnosis codes used to determine comorbid conditions are shown in eTable 1 in the Supplement.

Using VA clinic stop codes (ie, the location where patients receive outpatient care), the health care professional variables were the clinical site associated with the index AF diagnosis (eg, primary care, cardiology, emergency department, and pharmacy) and whether there was a clinical encounter with a cardiologist within 90 days of the index diagnosis (eTable 2 in the Supplement).^33^ The facility variables were the VA clinical site where the index diagnosis was recorded and facility type, categorized as VA medical center, primary care or multispecialty community-based outpatient clinic, and other. We also examined the frequency of VA primary care visits in the year prior to an index AF diagnosis, categorized as fewer than 2 or at least 2 primary care clinic visits at a VA facility.

### Statistical Analyses

Statistical analyses were conducted from December 1, 2019, to March 31, 2020. We compared baseline patient, health care professional, and facility characteristics across race/ethnicity groups using χ^2^ tests for all categorical variables. For all race/ethnicity groups, we compared the proportion of patients initiating any OAC (warfarin or DOAC therapy), and for OAC initiators, we compared the proportions using warfarin vs DOAC therapy.

We used mixed-effects logistic regression to model the adjusted odds of initiating any OAC within 90 days of the index AF diagnosis and initiating warfarin or DOAC among OAC initiators. To adjust for facility-level differences in OAC prescribing and/or VA formulary administration, all models incorporated a random effect for the VA clinical site where the index AF diagnosis was established. We incorporated patient, health care professional, and facility variables into these regression models based on their association with OAC initiation in prior literature and our assessment of multicollinearity in predicting the study outcomes.^2,30,36^

We constructed models for each OAC outcome using a 3-step process. In step 1, we added patient demographic and clinical covariates (ie, categorical age, year of index diagnosis, CHA_2_DS_2_VASc risk score, renal disease, liver disease, history of prior bleeding, use of medications predisposing to bleeding, and continuous body mass index). In step 2, we added the health care professional (ie, clinical site of index AF diagnosis, encounter with a cardiologist within 90 days, and frequency of VA primary care visits) and facility covariates. In step 3, we added patient socioeconomic covariates (ie, area deprivation index and VA priority group) to the model. For all modeling steps, we determined the adjusted odds ratios (aORs) and 95% CIs for race/ethnicity groups.

We did not include region or rurality in our models given their collinearity with the area deprivation index. A sensitivity analysis was performed replacing area deprivation index with region and rurality in the regression model, which yielded no difference in outcomes; therefore, these data are not presented. Additionally, we did not include sex as an individual baseline covariate because it is captured within the CHA_2_DS_2_VASc stroke risk score.

For all race/ethnicity groups, we examined the proportions initiating any OAC therapy and, among OAC initiators, the proportions using DOAC or warfarin therapy by year of index AF diagnosis. To determine time trends for the initiation of each therapy, we constructed logistic regression models adjusted for the above patient clinical (eg, CHA_2_DS_2_VASc stroke risk) and socioeconomic, health care professional, and facility factors, with main effects for calendar year of the AF index diagnosis and race/ethnicity. In addition, we incorporated a year by race/ethnicity group interaction term into these models to assess differences in time trends for OAC therapies by race/ethnicity. For all analyses, we used a 2-tailed *P* < .05 to define statistical significance and conducted analyses using SAS version 9.4 (SAS Institute Inc).

## Results

### Baseline Patient Characteristics

The final cohort comprised 111 666 patients, of whom 109 386 were men (98.0%), 2280 were women (2.0%), and 95 493 were White (85.5%) (Table 1), with a mean (SD) age of 72.9 (10.4) years. Other racial/ethnic groups in decreasing frequency were Black (9.2% [n = 10 238]), Hispanic (3.7% [n = 4088]), Asian (1.6% [n = 1295]), and AI/AN (0.5% [n = 552]). The majority of patients had a moderate (62.9% [n = 70 184]) or high (24.3% [n = 27 107]) stroke risk based on CHA_2_DS_2_VASc scores (Table 1).

**Table 1.** Comparison of Baseline Characteristics by Race/Ethnicity for Patients With Incident Atrial Fibrillation^a^^,^^b^

Characteristic	Overall (N = 111 666)	White (n = 95 493)	Black (n = 10 238)	Hispanic (n = 4088)	Asian (n = 1295)	AI/AN (n = 552)
**Demographic and socioeconomic characteristics**						
Age at diagnosis, y						
18-39	582 (0.5)	402 (0.4)	102 (1.0)	62 (1.5)	7 (0.5)	9 (1.6)
40-64	18 684 (16.7)	13 840 (14.5)	3625 (35.4)	822 (20.1)	276 (21.3)	121 (21.9)
65-74	47 095 (42.2)	40 991 (42.9)	3893 (38.0)	1466 (35.9)	480 (37.1)	265 (48.0)
75-84	28 564 (25.6)	25 395 (26.6)	1725 (16.8)	1048 (25.6)	287 (22.2)	109 (19.8)
Sex						
Men	109 386 (98.0)	93 628 (98.0)	9922 (96.9)	4032 (98.6)	1263 (97.5)	541 (98.0)
Women	2280 (2.0)	1865 (2.0)	316 (3.1)	56 (1.4)	32 (2.5)	11 (2.0)
Geographic region						
Midwest	27 177 (24.8)	24 817 (26.3)	1868 (18.4)	252 (7.0)	137 (11.1)	103 (19.2)
Northeast	16 790 (15.3)	15 176 (16.1)	1167 (11.5)	329 (9.1)	69 (5.6)	49 (9.1)
South	44 583 (40.6)	36 809 (39.1)	5961 (58.8)	1245 (34.4)	363 (29.3)	205 (38.2)
West	20 506 (18.7)	17 363 (18.4)	1125 (11.1)	1181 (32.6)	658 (53.1)	179 (33.4)
Outside US territories and DC^c^	691 (0.6)	55 (0.1)	9 (0.1)	615 (17.0)	12 (1.0)	0
Level of rurality						
Large metro	45 428 (41.4)	36 187 (38.4)	6215 (61.4)	2233 (61.7)	611 (49.6)	182 (34.0)
Small metro	39 602 (36.1)	34 929 (37.1)	2911 (28.7)	1132 (31.3)	445 (36.1)	185 (34.5)
Micropolitan	13 748 (12.5)	12 789 (13.6)	583 (5.8)	163 (4.5)	121 (9.8)	92 (17.2)
Rural	10 948 (10.0)	10 305 (10.9)	418 (4.1)	94 (2.6)	54 (4.4)	77 (14.4)
VA enrollment priority group^d^						
Groups 1-3	49 932 (46.2)	41 760 (45.1)	5240 (53.1)	1892 (49.1)	760 (59.9)	280 (52.5)
Group 4	2561 (2.4)	2060 (2.2)	299 (3.0)	172 (4.5)	20 (1.6)	10 (1.9)
Group 5	28 023 (26.0)	23 564 (25.5)	2854 (28.9)	1234 (32.0)	233 (18.4)	138 (25.9)
Group 6	3503 (3.2)	3240 (3.5)	150 (1.5)	64 (1.7)	34 (2.7)	15 (2.8)
Groups 7-8	24 083 (22.3)	21 954 (23.7)	1326 (13.4)	492 (12.8)	221 (17.4)	90 (16.9)
Area deprivation index (percentile)						
Quintile 1 (1-29)	21 570 (20.3)	18 869 (20.7)	1348 (13.8)	723 (20.8)	540 (45.7)	90 (17.9)
Quintile 2 (30-46)	21 032 (19.8)	18 748 (20.5)	1460 (15.0)	560 (16.1)	189 (16.0)	75 (14.9)
Quintile 3 (47-62)	21 439 (20.2)	19 093 (20.9)	1510 (15.5)	569 (16.4)	170 (14.4)	97 (19.3)
Quintile 4 (63-78)	20 974 (19.8)	18 229 (20.0)	1812 (18.6)	675 (19.4)	144 (12.2)	114 (22.7)
Quintile 5 (79-100)	21 215 (20.0)	16 376 (17.9)	3626 (37.2)	948 (27.3)	138 (11.7)	127 (25.2)
**Clinical characteristics**						
Medical comorbidities						
Congestive heart failure	18 212 (16.3)	14 439 (15.1)	2673 (26.1)	780 (19.1)	226 (17.5)	94 (17.0)
Hypertension	84 944 (76.1)	71 820 (75.2)	8514 (83.2)	3243 (79.3)	963 (74.4)	404 (73.2)
Diabetes	70 673 (63.3)	59 542 (62.4)	7180 (70.1)	2797 (68.4)	800 (61.8)	354 (64.1)
Vascular disease	46 599 (41.7)	39 625 (41.5)	4450 (43.5)	1786 (43.7)	509 (39.3)	229 (41.5)
Prior stroke	14 363 (12.9)	12 026 (12.6)	1549 (15.1)	566 (13.8)	161 (12.4)	61 (11.1)
History of bleeding	42 538 (38.1)	35 572 (37.3)	4568 (44.6)	1745 (42.7)	436 (33.7)	217 (39.3)
Liver disease	5174 (4.6)	4139 (4.3)	679 (6.6)	264 (6.5)	62 (4.8)	30 (5.4)
Renal disease	19 288 (17.3)	15 273 (16.0)	2818 (27.5)	845 (20.7)	263 (20.3)	89 (16.1)
Medications predisposing to bleeding	52 930 (47.4)	43 241 (45.3)	6406 (62.6)	2391 (58.5)	627 (48.4)	265 (48.0)
Body mass index^e^						
18.5 to <25	19 814 (17.9)	16 687 (17.6)	1965 (19.4)	789 (19.4)	305 (23.7)	68 (12.4)
25 to <30	37 951 (34.3)	32 733 (34.6)	3080 (30.4)	1483 (36.5)	487 (37.9)	168 (30.7)
30 to <35	28 845 (26.1)	24 863 (26.3)	2520 (24.9)	1009 (24.8)	281 (21.9)	172 (31.4)
35 to <40	14 327 (12.9)	12 253 (12.9)	1411 (13.9)	461 (11.3)	127 (9.9)	75 (13.7)
≥40	8927 (8.1)	7501 (7.9)	1011 (10.0)	280 (6.9)	75 (5.8)	60 (11.0)
CHA_2_DS_2_VASc stoke risk (score)^f^						
Low (0-1)	14 375 (12.9)	12 100 (12.7)	1488 (14.5)	501 (12.3)	188 (14.5)	98 (17.8)
Moderate (2-4)	70 184 (62.9)	60 664 (63.5)	5962 (58.2)	2434 (59.5)	786 (60.7)	338 (61.2)
High (>4)	27 107 (24.3)	22 729 (23.8)	2788 (27.2)	1153 (28.2)	321 (24.8)	116 (21.0)
Year of AF diagnosis						
2014	19 804 (17.7)	17 007 (85.9)	1769 (8.9)	698 (3.5)	222 (1.1)	108 (0.6)
2015	21 817 (19.5)	18 670 (85.6)	1942 (8.9)	848 (3.9)	267 (1.2)	90 (0.4)
2016	23 436 (21.0)	20 019 (85.4)	2155 (9.2)	854 (3.6)	279 (1.2)	129 (0.6)
2017	25 430 (22.8)	21 790 (85.7)	2305 (9.1)	929 (3.7)	284 (1.1)	122 (0.5)
2018	21 179 (19.0)	18 007 (85.0)	2067 (9.8)	759 (3.6)	243 (1.2)	103 (0.5)
≥2 VA primary care visits within year	84 213 (75.4)	71 329 (74.7)	8207 (80.2)	3328 (81.4)	936 (72.3)	413 (74.8)
**Health care professional and facility characteristics**						
Clinical site of diagnosing health care professional						
Primary care	61 696 (50.0)	53 867 (51.2)	4680 (41.2)	2072 (44.4)	761 (53.7)	316 (51.5)
Cardiology	18 035 (14.6)	14 786 (14.0)	2045 (18.0)	888 (19.0)	222 (15.7)	94 (15.3)
Emergency department	17 513 (14.2)	14 291 (13.6)	2088 (18.4)	884 (19.0)	156 (11.0)	94 (15.3)
Pharmacy	18 781 (15.2)	16 334 (15.5)	1683 (14.8)	511 (11.0)	172 (12.1)	81 (13.2)
Other	7293 (5.9)	5988 (5.7)	863 (7.6)	308 (6.6)	105 (7.4)	29 (4.7)
Cardiology visit within 90 d of AF	58 147 (52.1)	48 363 (50.6)	6237 (60.9)	2607 (63.8)	640 (49.4)	300 (54.3)
VA facility of AF diagnosis						
VAMC	73 316 (64.4)	61 357 (62.9)	7814 (75.4)	2948 (70.6)	831 (63.3)	366 (64.6)
Primary care CBOC	16 676 (14.6)	15 100 (15.5)	857 (8.3)	399 (9.6)	242 (18.4)	78 (13.8)
Multispecialty CBOC	16 925 (14.9)	15 138 (15.5)	969 (9.3)	572 (13.7)	159 (12.1)	87 (15.3)
Other	7009 (6.2)	5911 (6.1)	728 (7.0)	254 (6.1)	80 (6.1)	36 (6.3)

Abbreviations: AF, atrial fibrillation; AI/AN, American Indian/Alaska Native; CBOC, Community Based Outpatient Clinic; VA, Veterans Health Administration; VAMC, Veteran Affairs Medical Center.

^a^ With the exception of year of AF diagnosis (*P* = .09), all baseline characteristics differed significantly (*P* < .001) across race/ethnicity groups.

^b^ All percentages were calculated with missing data removed from the denominator. Data were missing for less than 5% of patients for region, rurality, area deprivation index, VA enrollment priority group, body mass index, and facility type and <0.5% for the remaining variables.

^c^ Represents veteran patients residing in US territories beyond the 50 states (eg, Guam, American Samoa, and Puerto Rico).

^d^ Priority groups convey veterans’ level of eligibility for VA services; lower groups have increased eligibility.

^e^ Calculated as weight in kilograms divided by height in meters squared.

^f^ CHA_2_DS_2_VASc indicates a score composed of points for congestive heart failure; hypertension; age ≥75 years; diabetes mellitus; prior stroke, transient ischemic attack, or thromboembolism; vascular disease; age 65-74 years; and sex category (female).

### Initiation of Any Anticoagulant Therapy by Race/Ethnicity

**Figure 2.**
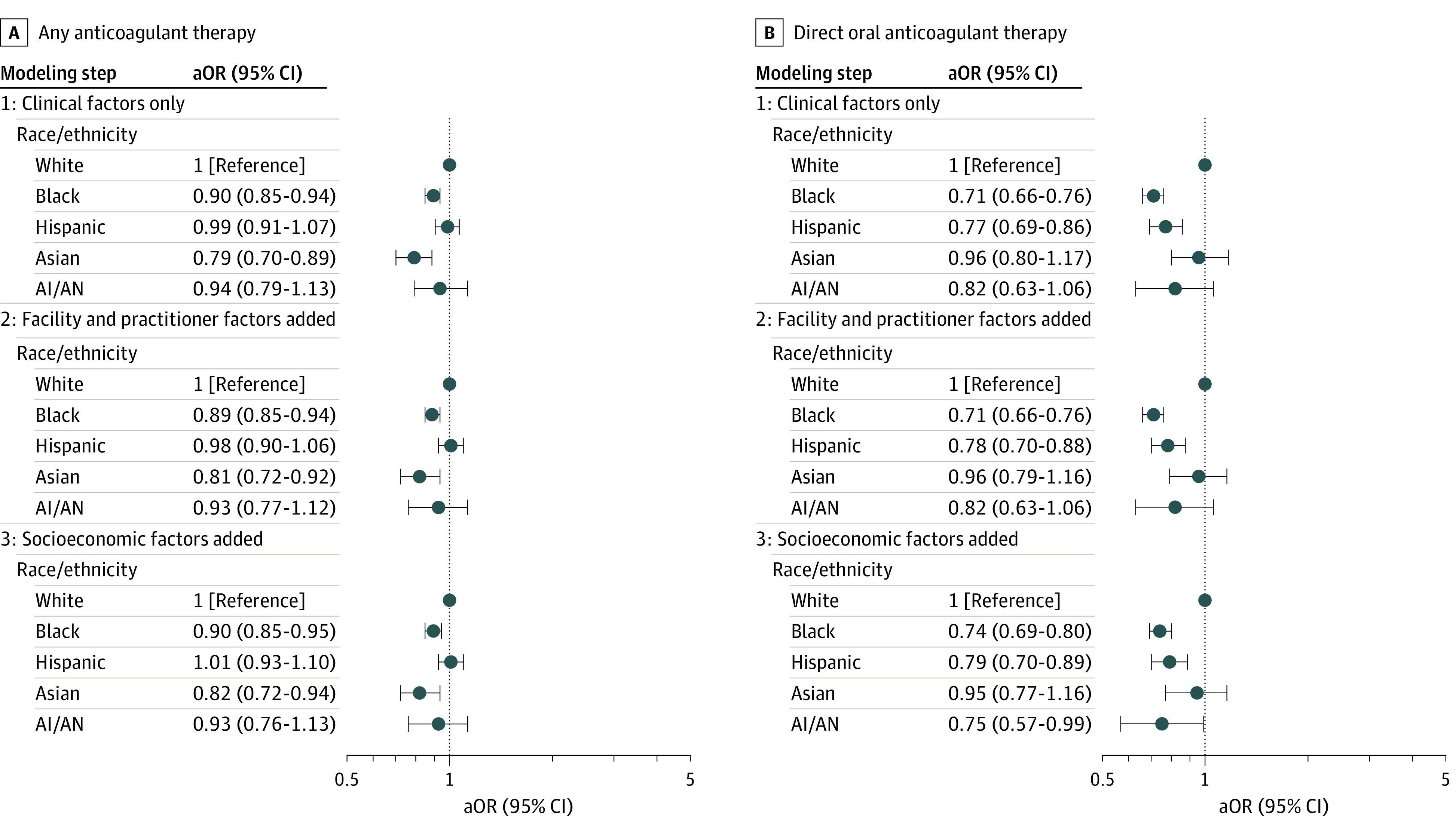
Adjusted Odds Ratios for Initiation of Any Anticoagulant Therapy and Direct Oral Anticoagulant (DOAC) Therapy by Race/Ethnicity for Patients With Incident Atrial Fibrillation Both analyses used a sequential logistic regression modeling approach considering patient demographic and clinical factors only (step 1), adding health care professional and facility factors (step 2), and adding patient socioeconomic factors (step 3). A, Black and Asian patients had significantly lower adjusted odds ratios (aORs) of receiving any oral anticoagulant therapy than White patients in the fully adjusted step 3 model. B, Black, Hispanic, and American Indian/Alaska Native (AI/AN) patients had significantly lower aORs of receiving DOAC therapy than White patients, with minimal attenuation in the magnitude of association across the 3 modeling steps.

Overall, 69 590 patients (62.3%) initiated any OAC therapy, varying 10.5 percentage points by race/ethnicity (*P* < .001); initiation was lowest in Asian (52.2% [n = 676]) and Black (60.3% [n = 6177]) patients and highest in White patients (62.7% [n = 59 881]) (Table 2). In our final model adjusting for all patient, health care professional, and facility factors, the adjusted odds of initiating any OAC therapy were significantly lower for Asian (aOR, 0.82; 95% CI, 0.72-0.94) and Black (aOR, 0.90; 95% CI 0.85-0.95) patients (Figure 2A). The magnitude of odds ratios changes between modeling steps by race/ethnicity are shown in Figure 2A, and the additional factors independently associated with any OAC initiation are shown in eTable 3 in the Supplement.

**Table 2.** Comparison of Initiation of Anticoagulant Therapy by Race and Ethnicity for Veterans With Incident Atrial Fibrillation and in Those Initiating Anticoagulant Therapy

Anticoagulant therapy	No. (%)						*P* value
	Overall	White	Black	Hispanic	Asian	AI/AN	
Any oral anticoagulant	69 590 (62.3)	59 881 (62.7)	6177 (60.3)	2520 2520 (61.6)	676 (52.2)	336 (60.8)	<.001
Warfarin^a^	24 214 (34.8)	20 384 (34.0)	2414 (39.1)	1050 (41.7)	231 (34.2)	135 (40.2)	<.001
Direct oral anticoagulant^a^	45 381 (65.2)	39 502 (66.0)	3763 (60.9)	1470 (58.3)	445 (65.8)	201 (59.8)	<.001

Abbreviation: AI/AN, American Indian/Alaska Native.

^a^ The denominators represented in these rows are individuals who initiated any oral anticoagulant therapy.

### Initiation of DOAC Therapy by Race/Ethnicity Among Anticoagulant Initiators

Among those who initiated any OAC therapy, 45 381 patients (65.2%) initiated DOACs (Table 2). The DOACs prescribed were apixaban (23 780 [52.4%]), dabigatran (9893 [21.8%]), rivaroxaban (11 209 [24.7%]), and edoxaban (499 [1.1%]). Initiation of DOACs varied by 7.7 percentage points across race/ethnicity groups (*P* < .001) and was lowest in Hispanic (1470 [58.3%]), AI/AN (201 [59.8%]), and Black (3763 [60.9%]) patients and highest in White patients (39 502 [66.0%]) (Table 2). In models adjusting for all patient, health care professional, and facility factors, the adjusted odds of initiating DOAC therapy were significantly lower for Hispanic (aOR, 0.79; 95% CI, 0.70-0.89), AI/AN (OR, 0.75; 95% CI, 0.57-0.99), and Black patients (aOR, 0.74; 95% CI, 0.69-0.80) (Figure 2B). The magnitude of odds ratio changes between modeling steps by race/ethnicity are shown in Figure 2B, and the additional factors independently associated with DOAC initiation are shown in eTable 4 in the Supplement.

### Trends in Anticoagulant Prescribing by Race/Ethnicity Over Time

**Figure 3.**
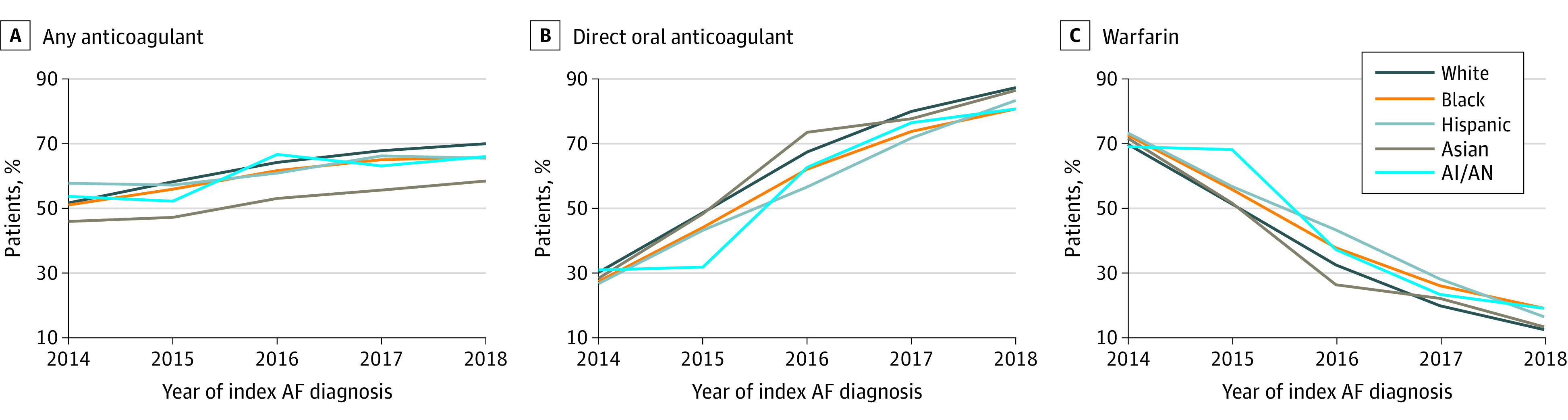
Time Trends for Initiating Any Anticoagulant, Direct Oral Anticoagulant, and Warfarin by Race/Ethnicity for Patients With Incident Atrial Fibrillation In logistic regression modeling with main effects for race/ethnicity and year and an interaction term for these variables, adjusted by patient clinical and socioeconomic, health care professional, and facility-level factors, there were statistically significant (*P* < .001) time trends of initiating any oral anticoagulant therapy and either a direct-acting oral anticoagulant or warfarin among anticoagulant initiators. There were no significant differences for the interaction of time and race/ethnicity over time for these anticoagulation outcomes.

From 2014 to 2018, initiation of any anticoagulant therapy increased from 52.0% to 65.1% overall (Figure 3). Among anticoagulant initiators, DOAC use increased from 29.9% to 86.7%, and warfarin use decreased from 70.1% to 13.3% (Figure 3). In models of any OAC initiation and DOAC use among initiators, adjusted for previously described patient clinical and socioeconomic, health care professional, and facility factors, main effects for race/ethnicity and year of index diagnosis were statistically significant (aOR, 1.19; 95% CI, 1.18-1.20 for any OAC initiation and aOR, 2.06; 95% CI, 2.03-2.09 for DOAC initiation). Notably, interaction terms for race/ethnicity and time were nonsignificant, indicating similar changes over time for any OAC and DOAC initiation in all race/ethnicity groups.

## Discussion

In a national cohort study of 111 666 patients with incident AF treated in the VA system from 2014 through 2018, Black and Asian patients were significantly less likely than White patients to initiate any OAC when adjusting for clinical, sociodemographic, physician, and facility factors. Among those who initiated OAC, Black, Hispanic, and AI/AN patients were significantly less likely to initiate DOACs. While overall OAC initiation and DOAC use increased significantly over time, there were no significant differences by race/ethnicity in the initiation of these treatments over time.

Using contemporary, real-world data from the largest integrated US health system, our findings extend upon prior non-VA studies that examined racial/ethnic disparities in anticoagulation for AF. In an analysis of the Outcomes Registry for Better Informed Treatment of Atrial Fibrillation II (ORBIT-AF II),^23^ Black patients were less likely than White patients to initiate any anticoagulant, a difference driven largely by lower DOAC use. Although the magnitude of racial differences in ORBIT-AF II were similar to our study, the analysis was limited to patient-level clinical and sociodemographic control factors. A recent American Heart Association Get with the Guidelines–Atrial Fibrillation analysis^44^ observed that Hispanic patients hospitalized with AF were less likely than White patients to be prescribed an OAC on discharge. Our observations of similarly low OAC initiation rates in Black and Hispanic patients, despite patients receiving care in a VA health system with improved access to medical therapy, suggests additional drivers of treatment disparities in AF beyond traditionally explored factors. Another VA-based observational study examined all prevalent AF cases between 2007 and 2016 and similarly observed that Black patients had lower odds of receiving DOAC compared with warfarin therapy.^3^ Our analysis examines both incident and confirmed cases of AF using a more contemporary patient cohort. Our cohort was able to capture the rapid update of DOACs as data on their superior effectiveness and safety become more accepted over time (ie, in 2017 and 2018), while adding to the literature on racial/ethnic differences in anticoagulation use for AF.

Our analysis also assessed Asian and AI/AN populations, which have been understudied in prior AF cohorts.^3^ One single-system study found that Asian patients were less likely than White patients to receive any OAC after adjusting for clinical and sociodemographic factors, with no difference seen in DOAC initiation.^45^ This disparity in OAC initiation was similar to our national observation. An analysis of the American College of Cardiology’s National Cardiovascular Data Registry database’s PINNACLE registry found that AI/AN patients were significantly less likely to be treated with OAC than non-AI/AN patients after controlling for similar factors.^46^ Notably, these analyses focused on an earlier time frame of DOAC use and did not control for many of the health care professional–level and facility-level factors incorporated into our analyses.

There are several mechanisms by which the observed racial/ethnic disparities may exist, particularly in DOAC use. At the patient level, research has demonstrated that minority patients may be less willing to accept novel therapeutics (eg, invasive cardiovascular procedures), which may carry over to medical management of AF.^47^ Decreased access to information about newer DOAC therapy through direct-to-consumer commercial advertisement may limit patient activation and shared decision-making related to anticoagulation.^48,49^ Additionally, a legacy of medical experimentation in racial/ethnic minority groups may limit trust in newer medical therapies in these communities.^50^

At the health care professional level, research has demonstrated worse access to medical specialists, including cardiologists, for minority patients.^51^ Our analysis suggests that referral to cardiology within 90 days of an index AF diagnosis is a driver for initiating anticoagulation (eTables 3 and 4 in the Supplement).^52^ Additionally, health care professional bias in prescribing might play a role. Previous literature has described over-attribution to adverse clinical and social risk factors in Black patients compared with White patients, which may result in differential prescribing.^53,54^ Finally, at the system level, while the impact of cost, particularly for higher-priced DOACs, should be lower in the VA system given its uniform drug formulary and low to negligible medication copayments, out-of-pocket costs associated with care access (eg, transportation, parking, and time off work) remain a key driver of treatment disparities.^55,56^ Health system guidelines to support improved documentation of when and how health care professionals offer prescriptions and reasons for patient refusal of anticoagulant therapy will be important to fully understand disparities in AF management.

### Limitations

There are limitations to our study. First, there may be concerns about the generalizability of an analysis of patients receiving care through the VA system. However, this study represents one of the largest national analyses of racial/ethnic minority groups with AF; apart from sex differences, the clinical factors closely resemble AF cohorts outside VA. Second, our assessment of socioeconomic factors relied on well-validated neighborhood-level characteristics but did not capture individual-level measures, such as income and educational level, or systemic factors, such as structural racism, that may perpetuate racial/ethnic disparities in treatment. Third, our analysis included a robust assessment of clinical factors, yet we were limited in our capture of possible contraindications to OAC initiation or patient treatment preferences. Fourth, we were unable to determine whether disparities in initiation of anticoagulation was related to differential health care professional prescribing or patient refusal of such therapy. Finally, our analysis was limited to a sample of patients receiving care within the VA system without assessment of medications received outside the VA from commercial or Medicare insurers. We suspect it is unlikely that adding non-VA medication data would eliminate the disparities identified here, as prior research has shown that White veterans are more likely to receive care outside the VA system compared with Black and Hispanic veterans.^36,57^ Nonetheless, future studies are needed to better understand the association of dual VA and Medicare eligibility on racial/ethnic disparities in access to anticoagulation for AF.^58^

## Conclusions

In a large national cohort of patients with AF treated in the VA health care system, we found lower initiation of any OAC and DOAC use in racial/ethnic minority groups, controlling for clinical and sociodemographic, provider, and facility-level factors. The persistence of disparities within the VA system has significant implications for ensuring equitable access to AF management within the broader US population. Implementing interventions to improve equity in anticoagulation initiation is critical for reducing disparities in this increasingly common and morbid condition.

## Notes

Supplement. **eTable 1.** ICD Codes to Define Conditions Analyzed **eTable 2.** Stop Codes to Define Primary Care, Cardiology, Pharmacy, Emergency Department, and Anticoagulant Clinic Usage **eTable 3.** Independent Associations of Patient, Provider, and Facility Characteristics and Initiation of Any Anticoagulant Therapy in Veterans with Atrial Fibrillation **eTable 4.** Independent Associations of Patient, Provider, and Facility Characteristics and Initiation of DOAC Therapy in Veterans with Atrial Fibrillation who Initiated Anticoagulant Therapy
