# Supplementary material for: Disparities in Anticoagulant Therapy Initiation for Incident Atrial Fibrillation by Race/Ethnicity Among Patients in the Veterans Health Administration System
Source: JAMA Netw Open. 2021 Jul 28;4(7):e2114234. doi: 10.1001/jamanetworkopen.2021.14234 (PMC8319757; doi:10.1001/jamanetworkopen.2021.14234)
Supplement: Supplement. — eTable 1. ICD Codes to Define Conditions Analyzed eTable 2. Stop Codes to Define Primary Care, Cardiology, Pharmacy, Emergency Department, and Anticoagulant Clinic Usage eTable 3. Independent Associations of Patient, Provider, and Facility Characteristics and Initiation of Any Anticoagulant Therapy in Veterans with Atrial Fibrillation eTable 4. Independent Associations of Patient, Provider, and Facility Characteristics and Initiation of DOAC Therapy in Veterans with Atrial Fibrillation who Initiated Anticoagulant Therapy [file jamanetwopen-e2114234-s001.pdf]

Supplemental Online Content

Essien UR, Kim N, Hausmann LRM, et al. Disparities in anticoagulant therapy initiation for incident atrial fibrillation by race/ethnicity among patients in the Veterans Health Administration system. *JAMA Netw Open*. 2021;4(7):e2114234. doi:10.1001/jamanetworkopen.2021.14234

eTable 1. ICD Codes to Define Conditions Analyzed

eTable 2. Stop Codes to Define Primary Care, Cardiology, Pharmacy, Emergency Department, and Anticoagulant Clinic Usage

eTable 3. Independent Associations of Patient, Provider, and Facility Characteristics and Initiation of Any Anticoagulant Therapy in Veterans with Atrial Fibrillation

eTable 4. Independent Associations of Patient, Provider, and Facility Characteristics and Initiation of DOAC Therapy in Veterans with Atrial Fibrillation Who Initiated Anticoagulant Therapy

This supplemental material has been provided by the authors to give readers additional information about their work.

© 2021 Essien UR et al. *JAMA Network Open.*

eTable 1. ICD Codes to Define Conditions Analyzed

Category Type	ICD-9-CM		ICD-10-CM	
Valvular Conditions (Mitral Valve and Aortic Valve)	395	Rheumatic aortic stenosis	I06.0	Rheumatic aortic stenosis
	395.1	Rheumatic aortic insufficiency	I06.1	Rheumatic aortic insufficiency
	395.2	Rheumatic aortic stenosis with insufficiency	I06.2	Rheumatic aortic stenosis with insufficiency
	395.9	Other and unspecified rheumatic aortic diseases	I06.8	Other rheumatic aortic valve diseases
	424.1	Aortic valve disorders	I06.9	Rheumatic aortic valve disease, unspecified
	394	Mitral stenosis	I08.2	Rheumatic disorders of both aortic and tricuspid valves
	394.1	Rheumatic mitral insufficiency	I35.0	Nonrheumatic aortic (valve) stenosis
	394.2	Mitral stenosis with insufficiency	I35.1	Nonrheumatic aortic (valve) insufficiency
	394.9	Other and unspecified mitral valve diseases	I35.2	Nonrheumatic aortic (valve) stenosis with insufficiency
	396	Mitral valve stenosis and aortic valve stenosis	I35.8	Other nonrheumatic aortic valve disorders
	396.1	Mitral valve stenosis and aortic valve insufficiency	I35.9	Nonrheumatic aortic valve disorder, unspecified
	396.2	Mitral valve insufficiency and aortic valve stenosis	Z95.2	Presence of prosthetic heart valve
	396.3	Mitral valve insufficiency and aortic valve insufficiency	Z95.3	Presence of xenogenic heart valve
	396.8	Multiple involvement of mitral and aortic valves	Z95.4	Presence of other heart-valve replacement
	396.9	Mitral and aortic valve diseases, unspecified	I05.0	Rheumatic mitral stenosis
	397.9	Rheumatic diseases of endocardium, valve unspecified	I05.1	Rheumatic mitral insufficiency
	424	Mitral valve disorders	I05.2	Rheumatic mitral stenosis with insufficiency
			I05.8	Other rheumatic mitral valve diseases
			I05.9	Rheumatic mitral valve disease, unspecified
			I08.0	Rheumatic disorders of both mitral and aortic valves
			I08.1	Rheumatic disorders of both mitral and tricuspid valves
			I08.3	Combined rheumatic disorders of mitral, aortic and tricuspid valves
			I08.8	Other rheumatic multiple valve diseases
			I08.9	Rheumatic multiple valve disease, unspecified
			I09.1	Rheumatic diseases of endocardium, valve unspecified
			I09.89	Other specified rheumatic heart diseases

© 2021 Essien UR et al. *JAMA Network Open.*

		I34.0 Nonrheumatic mitral (valve) insufficiency<br>I34.1 Nonrheumatic mitral (valve) prolapse<br>I34.2 Nonrheumatic mitral (valve) stenosis<br>I34.8 Other nonrheumatic mitral valve disorders<br>I34.9 Nonrheumatic mitral valve disorder, unspecified
Valvular Conditions – Procedural Code	35.05, 35.06, 35.2, 35.22, 35.23, 35.24	025F0ZZ, 025F3ZZ, 02QF0ZZ, 02QF3ZZ, 025G0ZZZ, 027G0ZZ, 02QG0ZZ, 02QG3ZZ
Atrial Fibrillation	427.31 Atrial fibrillation	I48.0 Paroxysmal atrial fibrillation<br>I48.1 Persistent atrial fibrillation<br>I48.2 Chronic atrial fibrillation<br>I48.91 Unspecified atrial fibrillation
Cardiac Ablation – Procedural Code	37.33, 37.34	02560ZZ, 02563ZZ, 02564ZZ, 02570ZZ, 02573ZZ, 02574ZZ
Hyperthyroidism	242, 242.81, 242.9x, 246.9	E05.00, E05.81, E05.9x, E07.9
Congestive Heart Failure	398.91, 402.xx, 404.01, 404.03, 404.1, 404.11, 404.13, 404.9, 404.91, 404.93, 428.xx,	I42.x, I50.xx,
Hypertension	401.x, 402.xx, 403.xx, 404.xx, 405.xx, 437.2	I10, I11.x, I12.x, I13.x, I15.x, I16.x, I50.30, I50.40, I50.9, N03.9, N18.x, N19, Z99.2
Diabetes	249.xx, 250.xx, 357.2, 362.xx, 366.41,	B35.1, E03.9, E08.xxxx, E09.xxxx, E10.xxxx, E11.xxxx, E13.xxxxx,E23.2, E27.49, E66.9, E78.1, E78.6, G56.00, H21.1X9, H33.4, H34.9, H35.049, H40.9, H42, H43.1, H47.099, H47.2, H54.0, H54.10, H54.7, H91.9, I10, I12.x, I70.209, K31.84, L03.039, L03.119, L89.509, L89.609, L97.209, L97.309, L97.409, L97.509, L97.519, L97.529, L97.909, L97.919, L97.929, L98.499, M54.14, M54.16, M86.9, N18.1, N18.2, N18.3, N18.4, N18.5, N18.6, N18.9, N52.1, R19.7, R80.9, Z79.4, Z99.2
Vascular Disease	410.xx, 411.xx, 412, 429.79, 440.xx, 441.xx, 442.xx, 443.xx, 444.xx, 445.xx	E11.9, G40.909, H91.90, I20.x, I21.xx, I23.x, I24.x, I25.xxx, I51.0, I51.1, I51.2, I51.3, I51.4, I51.5, I51.9, I70.xxx, I71.xx, I72.x, I73.xx, I74.xx, I75.xxx, N28.9

© 2021 Essien UR et al. *JAMA Network Open.*

Prior Ischemic Stroke	433.xx, 434.xx, 435.x, 436, 437.xx, 438.xx, v12.54	G45.xx, G93.1, G93.2. G93.49, I63.xxx, I65.xx, I66.xx I67.xxx, I68.xx, I69.0xx, I69.xxx, I74.xx, Z86.73
Prior Bleeding	280.x, 281.xx, 282.xx,, 283.xx,, 284.xx, 285.xx, 423.0, 430, 431, 432.x, 455.x, 456..0, 456.2x, 459.0, 530.7, 530.8x, 531.xx, 532, 532.2x, 532.4x, 532.6x, 533.2x, 533.4x, 534.4x, 535.01, 535.11, 535.31, 535.41, 535.51, 535.61, 537.8x, 562.02, 562.03, 562.12, 562.13, 568.xx, 569.3, 569.85, 578.x, 599.7x, 719.1x, 782.7, 784.7, 784.8, 786.3x,	D50.x, D51.x, D52.x, D53.x, D55.x, D56.x, D57.xxx, D58.x, D59.x, D60.x, D61.xxx, D62, D63.x, D64.xx, I31.2, I60.xx, I61.x, I62.xx, I85.x, K22.xx, K25.2, K25.4, K25.6, K25.0, K26.2, K26.4, K26.6, K27.0, K27.2, K27.4, K27.6, K28.0, K28.2, K28.4, K28.6, K29.01, K29.21, K29.31, K29.41, K29.51, K29.61, K.71, K29.81, K29.91, K31.811, K31.82, K55.2x, K57.01, K57.11, K57.13, K57.21, K57.31, K57.33, K57.41, K57.51, K57.53, K57.81, K57.91, K57.93, K62.5, K64.x, K66.1, K92.0, K92.1, K92.2, M25.0xx, N32.xx, R04.xx, R23.3, R31.xx, R58.0
Liver Disease	570, 571.xx, 572.x, 573.x	K70.xx, K71.xx, K72.xx, K73.x, K74.xx, K75.xx, K76.xx, K77
Renal Disease	582.xx, 583.xx, 585.x, 586, 790.4	N03.x, N05.x, N08, N17.x, N18.xx, N19, R74.0

© 2021 Essien UR et al. *JAMA Network Open.*

eTable 2. Stop Codes to Define Primary Care, Cardiology, Pharmacy, Emergency Department, and Anticoagulant Clinic Usage

Service Line	Stop Codes
Primary Care	322, 323, 348, 350, 704, 301, 170, 171, 338
Cardiology	303
Emergency Department	130, 131
Pharmacy	160, 317, 338, 176
Anticoagulant Clinic	317

© 2021 Essien UR et al. *JAMA Network Open.*

eTable 3. Independent Associations of Patient, Provider, and Facility Characteristics and Initiation of Any Anticoagulant Therapy in Veterans with Atrial Fibrillation

Type of Characteristics	Adjusted Odds Ratio	95% CI
**Demographic and socioeconomic characteristics**		
Race		
White	**Reference**	—
Black	0.90	0.85-0.95
Hispanic	1.01	0.93-1.10
Asian	0.82	0.72-0.94
American Indian / Alaska Native	0.93	0.76-1.13
Age at diagnosis (years)		
18-39	0.20	0.16-0.24
40-64	0.69	0.66-0.72
65-74	**Reference**	—
≥75-84	0.87	0.84-0.91
85+	0.57	0.54-0.60
VA enrollment priority group		
Groups 1 - 3	**Reference**	—
Group 4	0.72	0.66-0.79
Group 5	1.14	1.10-1.18
Group 6	1.18	1.08-1.28
Groups 7 - 8	1.34	1.29-1.39
Area deprivation index (percentile)		
Quintile 1 (1 - 29)	**Reference**	—
Quintile 2 (30 - 46)	0.99	0.95-1.04
Quintile 3 (47 - 62)	1.01	0.97-1.06
Quintile 4 (63 - 78)	1.02	0.98-1.07
Quintile 5 (79 - 100)	1.03	0.98-1.08
Rurality		
Large Metro	**Reference**	
Small metro	1.01	0.97-1.05
Noncore rural	1.10	1.03-1.16
Micropolitan	0.05	0.99-1.10
Region		
South	**Reference**	
Midwest	1.14	1.07-1.22
Northeast	0.91	0.85-0.98
West	0.89	0.82-0.95
Outside of 50 states	1.09	0.76-1.55

© 2021 Essien UR et al. *JAMA Network Open.*

Type of Characteristics	Adjusted Odds Ratio	95% CI
**Clinical characteristics**		
Medical comorbidities		
History of bleeding	0.82	0.79-0.84
Liver disease	0.65	0.61-0.69
Renal disease	0.82	0.79-0.85
Medications predisposing to bleeding	1.13	1.09-1.16
Body mass index (Kg/M^2)		
<18.5	0.47	0.40-0.55
18.5 - <25	0.76	0.73-0.79
25 - <30	**Reference**	—
30 - <35	1.14	1.10-1.18
35 – <40	1.32	1.26-1.39
≥40	1.40	1.32-1.48
CHA2DS2-VASc stoke risk (score)		
Low (0-1)	**Reference**	—
Moderate (2-4)	1.76	1.68-1.84
High (>4)	1.86	1.75-1.97
Year of AF diagnosis		
2014	**Reference**	—
2015	1.27	1.21-1.33
2016	1.56	1.49-1.63
2017	1.80	1.72-1.88
2018	2.03	1.94-2.12
≥2 VA primary care visits within year*	1.11	1.07-1.15
**Provider and facility characteristics**		
Clinical site of diagnosing provider		
Primary care	**Reference**	—
Cardiology	0.78	0.74-0.81
Emergency department	1.46	1.39-1.53
Pharmacy	6.60	6.20-7.04
Other	0.63	0.60-0.67
Cardiology visit ≤90 days of AF diagnosis	1.97	1.91-2.03
VA facility type of AF diagnosis		
VAMC†	**Reference**	—
Primary care CBOC‡	0.96	0.89-1.03
Multi-specialty CBOC‡	0.99	0.91-1.07
Other	0.93	0.83-1.04

* VA= Veterans Health Administration
† VAMC = Veterans Affairs Medical Center
‡ CBOC = Community-Based Outpatient Clinics

© 2021 Essien UR et al. *JAMA Network Open.*

eTable 4. Independent Associations of Patient, Provider, and Facility Characteristics and Initiation of DOAC Therapy in Veterans with Atrial Fibrillation who Initiated Anticoagulant Therapy

Type of Characteristics	Adjusted Odds Ratio	95% CI
**Demographic and socioeconomic characteristics**		
Race		
White	**Reference**	—
Black	0.74	0.69-0.80
Hispanic	0.79	0.70-0.89
Asian	0.95	0.77-1.16
American Indian / Alaska Native	0.75	0.57-0.99
Age at diagnosis (years)		
18-39	2.23	1.42-3.51
40-64	0.98	0.92-1.04
65-74	**Reference**	—
≥75-84	1.19	1.12-1.25
85+	1.20	1.12-1.29
VA enrollment priority group		
Groups 1 - 3	**Reference**	—
Group 4	0.85	0.74-0.99
Group 5	0.83	0.79-0.87
Group 6	1.13	1.01-1.27
Groups 7 - 8	1.16	1.10-1.22
Area deprivation index (percentile)		
Quintile 1 (1 - 29)	**Reference**	—
Quintile 2 (30 - 46)	0.88	0.82-0.94
Quintile 3 (47 - 62)	0.83	0.77-0.89
Quintile 4 (63 - 78)	0.80	0.75-0.86
Quintile 5 (79 - 100)	0.75	0.70-0.80
Rurality		
Large Metro	**Reference**	
Small metro	0.94	0.88–1.00
Noncore rural	1.05	0.97-1.14
Micropolitan	0.96	0.89-1.04
Region		
South	**Reference**	
Midwest	0.85	0.75-0.95
Northeast	1.00	0.86-1.16
West	0.87	0.75-1.00
Outside of 50 states	0.70	0.37-1.31
**Clinical characteristics**		
Medical comorbidities		
History of bleeding	0.84	0.81-0.88

© 2021 Essien UR et al. *JAMA Network Open.*

Type of Characteristics	Adjusted Odds Ratio	95% CI
Liver disease	0.71	0.65-0.78
Renal disease	0.51	0.48-0.53
Medications predisposing to bleeding	1.04	1.00-1.08
Body mass index (Kg/M^2)		
<18.5	0.76	0.58-0.98
18.5 - <25	0.91	0.85-0.96
25 - <30	**Reference**	—
30 - <35	0.97	0.93-1.03
35 – <40	0.88	0.83-0.94
≥40	0.66	0.61-0.71
CHA2DS2-VASc stoke risk (score)		
Low (0-1)	**Reference**	—
Moderate (2-4)	0.98	0.91-1.06
High (>4)	0.84	0.76-0.91
Year of AF diagnosis		
2014	**Reference**	—
2015	2.38	2.23-2.53
2016	5.82	5.46-6.20
2017	12.06	11.28-12.90
2018	22.34	20.72-24.09
≥2 VA primary care visits within year*	0.93	0.88-0.98
**Provider and facility characteristics**		
Clinical site of diagnosing provider		
Primary care	**Reference**	—
Cardiology	0.95	0.89-1.02
Emergency department	0.87	0.82-0.93
Pharmacy	1.24	1.16-1.32
Other	0.74	0.67-0.81
Cardiology visit ≤90 days of AF diagnosis	0.58	0.56-0.61
VA facility type of AF diagnosis		
VAMC†	**Reference**	—
Primary care CBOC‡	0.97	0.83-1.13
Multi-specialty CBOC‡	0.95	0.80-1.13
Other	0.92	0.72-1.17

* VA= Veterans Health Administration
† VAMC = Veterans Affairs Medical Center
‡ CBOC = Community-Based Outpatient Clinics

© 2021 Essien UR et al. *JAMA Network Open.*
